# Influence of statistical approaches on Probabilistic Sweet Spots computation in Deep Brain Stimulation for severe Essential Tremor

**DOI:** 10.1016/j.nicl.2025.103820

**Published:** 2025-06-07

**Authors:** Vittoria Bucciarelli, Dorian Vogel, Teresa Nordin, Marc Stawiski, Jérôme Coste, Jean-Jacques Lemaire, Raphael Guzman, Simone Hemm

**Affiliations:** a Institute for Medical Engineering and Medical Informatics, School of Life Sciences, University of Applied Sciences and Arts Northwestern Switzerland FHNW, Hofackerstrasse 30, Muttenz, Switzerland; b Department of Biomedical Engineering, University of Basel, Allschwil, Switzerland; c Department of Biomedical Engineering, Linköping University, Linköping, Sweden; d Université Clermont Auvergne, CNRS, CHU Clermont-Ferrand, Clermont Auvergne INP, Institut Pascal, Clermont-Ferrand, France; e Department of Neurosurgery, University Hospital Basel, Basel, Switzerland

**Keywords:** Deep Brain Stimulation (DBS), Probabilistic Sweet Spot, Essential Tremor, Probabilistic mapping, Statistical methods

## Abstract

Deep Brain Stimulation (DBS) is an established therapy for movement and neuropsychiatric disorders. Identifying brain regions (Probabilistic Sweet Spots, PSS) linked with the greatest symptom improvement is crucial for refining pre-operative targeting and post-operative programming. Probabilistic stimulation mapping is a powerful data-driven tool to delineate these regions. However, the chosen statistical methods can influence the identified PSS. A comprehensive evaluation of their impact is lacking in DBS research. The present study compares the PSS generated with four voxel-wise statistical approaches — *t*-test, Wilcoxon test, Linear Mixed Model, and Bayesian *t*-test — with the aim of assessing their influence on computed results on the same dataset. Intra-operative stimulation test data of 23 Essential Tremor (ET) patients was used to run patient-specific electric field simulations and to generate PSS in a group-specific anatomical template space. The PSS for the different statistical tests were first compared in terms of size and topography. Then, their correlation with clinical improvement was calculated in a leave-one-out cross-validation scheme and PSS consistency across datasets with different compositions was assessed. Our findings emphasize the impact of statistical test selection on both the anatomical location and volume of the extracted PSS, highlighting the importance of careful methodological choices in future DBS mapping studies. The Bayesian *t*-test and a voxel-wise application of nonparametric permutation testing, introduced for the first time in DBS research, showed promising results in identifying PSS representative of improvement and exhibited robustness to variations in the dataset.

## Highlights

• Statistical method choice greatly impacts extracted probabilistic volumes in DBS.
• Bayesian t-tests offers robust, promising results for probabilistic mapping studies.
• Intra-operative stimulation tests provide valuable data for probabilistic mapping.

## Graphical abstract

**Figure:**
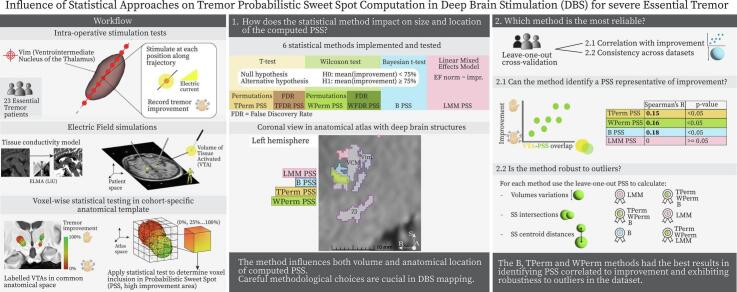

## 1 Introduction

Deep Brain Stimulation (DBS) is an established procedure to treat symptoms of severe movement and neuropsychiatric disorders (Benabid et al., 2000, Wårdell et al., 2022). The principle is based on understanding the structures involved in symptom pathophysiology, which historically showed significant symptom alleviation following target lesioning (Talairach et al. 1957). This concept of target was translated for the implantation of DBS electrodes delivering electrical currents in the surrounding tissues (Hemm and Wårdell 2010). In DBS surgery, beyond the technical challenges of target identification and electrode placement, there is a need for statistical analysis of stimulation effects in relation to brain structures. This is essential to gain a deeper understanding of the underlying therapeutic mechanisms (Lozano et al. 2019).

Moreover, identifying brain areas whose stimulation leads to high symptom improvement provides valuable information to guide pre-operative targeting and algorithms for post-operative parameter programming (Nordenström et al. 2022). The introduction of the Volume of Tissue Activated (VTA) concept enables the representation of a model volume where neuron bodies and axons should be directly modulated (Maks et al. 2009). Nevertheless, it is important to note that the clinical effects primarily depend on the underlying circuitry which is now indirectly accessible through structural and functional connectomics approaches (Schmitt et al., 2016, Bower et al., 2024). Individualized analysis of VTA-related effects can be extracted from intra-operative stimulation tests (Shah et al. 2020) and post-operative effective contact (e.g. used for chronic stimulation) tests (Nguyen et al., 2019, Dembek et al., 2017). Group level analysis of VTAs and related therapeutic effects from several patients leads to the generation of Probabilistic Stimulation Maps (PSMs) with data-driven methods (Eisenstein et al., 2014, Nguyen et al., 2019, Dembek et al., 2017).

The spread of current in the tissues depends on tissue properties, electrode location, and stimulation settings (Butson et al. 2010). Therefore, the input data to generate PSMs usually consists of patient brain images, implanted electrode locations, stimulation settings, and corresponding clinical outcomes (Dembek et al. 2022). Electric Field (EF) modelling is then performed with Finite Element Methods (FEM) to estimate the VTA by specific stimulation configurations (Åström et al. 2015). Each VTA, labelled with a specific effect (e.g. motor improvement score), is transformed to a standardized anatomical space such as the MNI152 (Fonov et al. 2011), Morel’s atlas (Morel 2007) or a group-specific template (Vogel et al. 2021). This operation allows the integration of data from multiple patients and constitutes the basis of group analysis (Wårdell et al. 2022). Each voxel in the common reference space is covered by multiple individual VTAs and is thus associated with a distribution of improvement scores. In the context of Deep Brain Stimulation (DBS) probabilistic mapping, a key objective is identifying “Probabilistic Sweet Spots” (PSS) by studying the improvement scores associated to each voxel. PSS, referred to in the literature as “effective stimulation regions” (Nordenström et al. 2022), “optimal stimulation regions” (Dembek et al. 2022), “probabilistic clinically effective stimulation sites” (Nguyen et al. 2019), or “volumes of high likelihood of favorable outcomes” (Reich et al. 2019), represent areas where stimulation yields positive therapeutic effects. For this work, the term “Probabilistic Sweet Spot” will denote brain volumes linked to good or excellent symptom improvement, as determined through probabilistic analyses of stimulation test data (excluding stimulations that caused side effects). Several criteria have been proposed to evaluate the membership of each voxel to the PSS. For example, the inclusion of voxels with an average clinical improvement higher than a specified threshold (Butson et al., 2010, Cooper et al., 2014, Johnson et al., 2019) or the selection of voxels activated in a minimum percentage of patients showing a high symptom improvement (Krishna et al., 2016, Cheung et al., 2014, Schaper et al., 2020). A popular and promising strategy is applying voxel-wise statistical testing to pinpoint voxels significantly associated with high symptom improvement. T-tests and Wilcoxon signed rank tests are among the most extensively adopted methods for such purpose (Nguyen et al., 2019, Reich et al., 2019, Treu et al., 2020, Elias et al., 2021, Al-Fatly et al., 2023, Kübler et al., 2021). Some studies have also used Linear Mixed Models (Vogel et al., 2024, Dembek et al., 2019) which account for variability across patients. The statistical testing assigns a p-value to each voxel, on the basis of which, the voxel is classified as belonging to the PSS or not. Given the high number of tests, type I error correction is performed by employing False Discovery Rate (FDR) (Neudorfer et al., 2021, Neudorfer et al., 2022, Nowacki et al., 2022, Nowacki et al., 2020), where p-values are ranked and compared to a threshold accounting for the desired rate of false discoveries (Benjamini and Hochberg 1995). Another possibility is applying nonparametric permutation analyses, by shuffling the data labels and creating a distribution of outcomes under the null hypothesis (Tödt et al., 2022, Petry-Schmelzer et al., 2019, Akram et al., 2017, Eisenstein et al., 2014, Dembek et al., 2019). The resulting PSS volumes and locations are highly dependent on the chosen mapping strategy, as shown by recent studies (Dembek et al., 2022, Nordin et al., 2022).

A thorough assessment of the different statistical tests and type I error correction and their influence on the computed PSS is, to the best of our knowledge, still missing in the literature. The objective of this study was to analyse the impact of the applied statistical methods on the identification of PSS and, if possible, determine the most reliable method for PSS computation. Thus, a comparison of PSS generated with four voxel-wise statistical methods (*t*-test, Wilcoxon test, Linear Mixed Model, Bayesian *t*-test) and two type I error corrections (FDR, nonparametric permutations) was performed. Bayesian statistics has shown promising results in studies analyzing fMRI data (Kang et al., 2011, Han and Park, 2019), but its application to the probabilistic mapping problem is a novelty in the DBS domain. This work also presents a new way of applying the concept of nonparametric permutations, providing a score on a voxel-wise basis rather than for the whole map, inspired by (Nichols and Holmes 2001). The size and topographical location of the PSS were used as initial criteria for comparison. However, it is important to emphasize that precise anatomical localization of the PSS is outside the scope of this work, which primarily focuses on the comparative evaluation of statistical methods. Subsequently, we evaluated whether the PSS were representative of improvement and assessed the consistency of the methods when presented to outliers, to identify the most suitable approach for determining PSS in DBS.

## 2 Materials and methods

### 2.1 Clinical data

#### 2.1.1 Patients

Intra-operative stimulation data of 23 patients was analysed. The data was collected during the exploration of Vim (ventrointermediate nucleus of the thalamus) for severe Essential Tremor DBS at the Department of Neurosurgery of Clermont-Ferrand University Hospital, France. The electrodes were implanted bilaterally, under local anesthesia. The first electrode was systematically positioned in the left hemisphere. A group-specific anatomical template (Vogel et al. 2021) was created from pre-operative inversion-recovery MR images (White Matter Inversion-Recovery sequence, WAIR) of 67 patients aged between 22 and 81. The patient cohort included in the template were the 23 ET patients and additionally 44 Parkinson’s Disease patients, all operated in the same institution between 2008 and 2018. The anatomical template was generated with an optimized anatomical normalization method described in (Vogel et al. 2020; 2021). The detailed implantation protocol can be found in (Vogel et al. 2024). All the patients signed a written informed consent (Ptolemee Electrophysiologie project: IRB 5921, CE-CIC-GREN-18–03) for the retrospective analysis of data. Patients’ clinical and imaging data was digitalized and handled using the data management system described in (Stawiski et al. 2024).

#### 2.1.2 Stimulation data

Intra-operative stimulation data consisted of improvement scores, stimulation current amplitude and location of the stimulation point. Stimulation (semi-electrode; Alpha-Omega Engineering, Israel; frequency 130 Hz; pulse width 60 µs) was applied over 14 mm with 1 mm step on 2 parallel tracts (2 mm away). The current amplitude was incremented from 0.2 mA to 3 mA (0.2 mA step). Tremor improvement was assessed by the same, experienced neurosurgeon according to a subjective 5-point rating scale (0 %: no improvement, 25 %: poor improvement, 50 %: fair improvement, 75 %: good improvement, 100 %: excellent improvement) and sometimes intermediate scores, if warranted by the evaluation. Improvement score of 100 % was assigned only upon complete tremor disappearance. For each test, tremor severity was evaluated relative to the patient’s baseline condition. The latter was assessed during the few seconds immediately preceding stimulation onset.

**Fig. 1:**
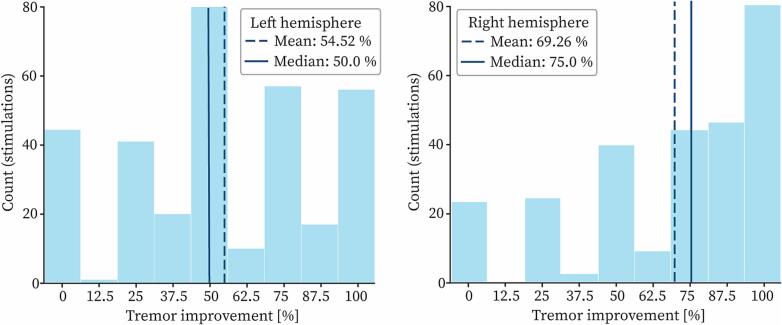
Tremor improvement values distributions: improvement scores for the left hemisphere are on the left and the ones for the right hemisphere on the right. The dotted line indicates the mean of the distribution and the full line the median. Count indicates the number of stimulations associated with a certain improvement value.

Motor thresholds (lowest current amplitude at which the best symptom improvement was observed) were recorded with their corresponding improvement score, resulting in one improvement score per position. Fig. 1 shows the improvement values distribution for the left and right hemisphere. Patients' symptoms were markedly reduced during the evaluation of the second hemisphere (right hemisphere in this study), likely as a consequence of the implantation of the first electrode. This led to a greater likelihood of achieving complete tremor suppression, resulting in improvement scores that were skewed toward higher values. Furthermore, patient fatigue following several hours of surgery allowed for a more meticulous exploration of the first implanted hemisphere. For these reasons, only stimulation data from the left hemisphere was used for the comparison of PSS in this study. An overview of the stimulation data is provided in Table 1.

**Table 1:** Overview of intra-operative stimulation data.

	Left hemisphere	Right hemisphere
Number of stimulations	326	244
Number of stimulations per patient (mean ± std)	15 ± 6	13 ± 4
Position range − distance to target (mm)	−10 to +4	−10 to +4
Amplitude range (mA)	0.2 to 5.0	0.2 to 3.4
Tremor improvement % (mean ± std)	54.52 ± 32.04	69.26 ± 31.61

### 2.2 Electric field simulations and anatomical normalization

**Fig. 2:**
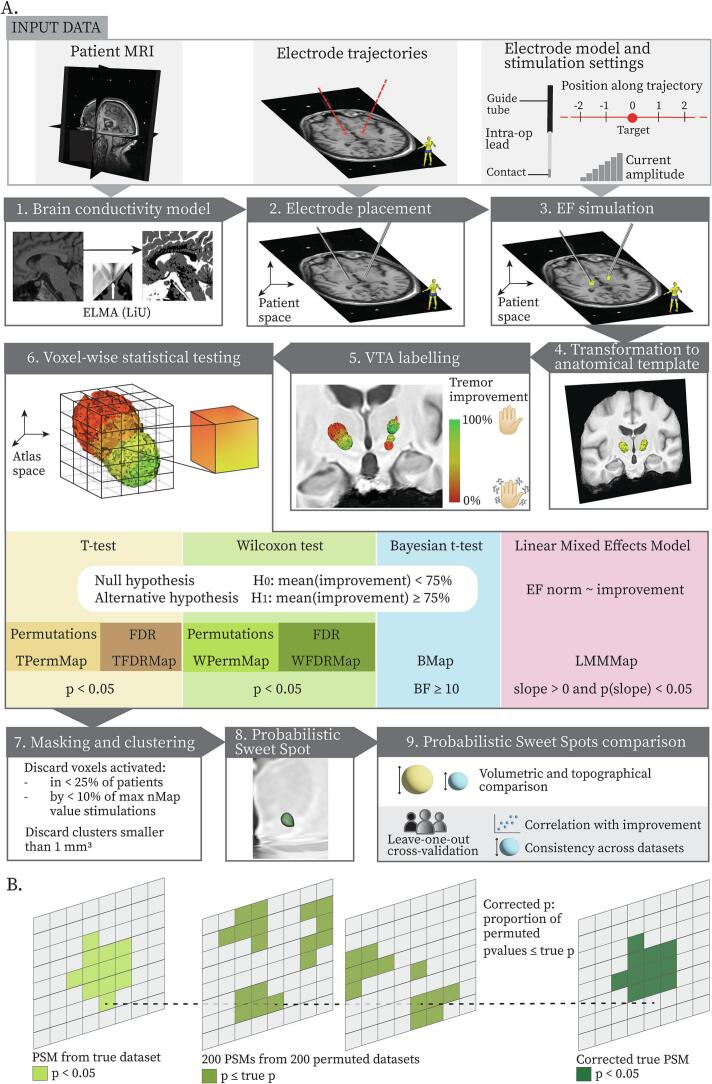
(A) Probabilistic Sweet Spot generation workflow. The workflow starts from input data collected pre- and intra-operatively (light grey background). For each patient a brain conductivity model was generated (1), and electrodes were placed in the patient reference space (2) to be able to run Electric Field (EF) simulations (3). The EFs were transformed to a group-specific anatomical template (4) so that data from the entire patient cohort could be collectively analyzed. The EFs were thresholded at 0.2 V mm^−1^ to generate the Volume of Tissue Activated (VTAs) and each VTA was labelled with the corresponding tremor improvement score (5). Voxel-wise statistical testing was performed to generate a PSM (6). In this work four statistical methods were applied: *T*-test, Wilcoxon test, Bayesian *t*-test and Linear Mixed Effects Model. The first two were corrected for type I error with a False Discovery Rate approach and a voxel-wise non-parametric permutation approach. As a consequence, six distinct PSMs were obtained. Voxels significantly associated with stimulation-induced symptom improvement were extracted by thresholding the PSM with a suitable threshold according to the statistical method. Those voxels were masked to discard the ones stimulated too few times or in too few patients (7), leading to the final Probabilistic Sweet Spot (8). The volumes, topography, correlation with improvement and geometric consistency across datasets of the 6 PSS were compared. These last two metrics were computed in a leave-one-out cross-validation fashion (9). (B) Graphical representation of the voxel-wise permutation correction. The dotted line shows how a voxel’s p-value in the original map is compared with its p-values in the permuted maps, to calculate the corrected p-value associated with that voxel. If the corrected p-value is ≥0.05 the voxel is discarded from the corrected PSS. The correction mechanism was adapted from (Nichols and Holmes 2001).

The workflow for generating patient-specific EF simulations and normalizing them in a reference anatomical space (group-specific template in this study) is described in detail in (Vogel et al. 2024) and shown in Fig. 2**A**. In brief, EF volumes were simulated following the modelling method described in (Åström et al. 2015). A patient-specific brain tissue conductivity model was obtained by segmenting white, grey matter and cerebrospinal fluid on the T1-weighted patient image using the Matlab-based, freely available ELMA software^1^ (Johansson et al., 2019, Wårdell et al., 2012). An affine transform allowed placing the electrode model in the patient reference space, using the 3D Slicer^2^ (Fedorov et al. 2012) plugin Stereotaxia (Beninca et al. 2017). Once the electrode was placed, Comsol Multiphysics 5.5 (COMSOL AB, Sweden) was used as the simulation environment to run FEM simulations of the EF spread in the tissue. The EF meshes for each patient and each stimulation configuration were transferred to the group-specific anatomical template by applying the appropriate coordinate transformations. The meshes were then resampled to a rectilinear grid, obtaining a NifTI image file for each EF simulation. The file was a volumetric image with the same resolution as the template (0.5x0.5x0.5 mm^3^), where each voxel contained the EF norm.

### 2.3 Voxel-wise statistical testing and type I error correction

Simulated EF labelled with the respective improvement scores were stacked as a 4D matrix in the group-specific template space. For each voxel, the first 3 dimensions corresponded to the x, y, and z coordinates, while the fourth dimension to the pairs [EF values, improvement scores]. Improvement scores associated with EF absolute values lower than 0.2 V mm^−1^ were discarded since this threshold is required to activate neurons with axon diameters of approximately 3 to 4 µm at a stimulation pulse width of 60 µs (Åström et al. 2015). Voxel-wise statistical testing was performed along the fourth dimension of the matrix to obtain 3D PSMs. Four statistical methods were implemented to obtain the significant maps: *t*-test (TMap), Wilcoxon test (WMap), Bayesian *t*-test (BMap) and a Linear Mixed Model (LMMMap). The *t*-test and Wilcoxon test were corrected for type I error inflation due to multiple comparisons. The computation workflow leading to the PSMs was run on a 32-core workstation with 128 GB of RAM. The statistical tests and data analysis were implemented in Python. The details related to each method are reported in the following sections.

#### 2.3.1 T-test and Wilcoxon test

The TMap and WMap were computed by applying two commonly used methods in frequentist hypothesis testing: one-sample *t*-test and one-sample Wilcoxon signed-rank test*.* Both tests were one-sided, with null hypothesis H_0_: improvement < threshold and alternative hypothesis H_1_: improvement ≥ threshold. Following clinical recommendations, a threshold of 75 % improvement was selected to identify voxels significantly associated with at least a “good” level of clinical benefit in line with the rating scale. The significance level was chosen to be α = 0.05. This value was used to threshold the TMap and WMap in order to obtain binary sweet spots formed by significant voxels (voxels with p-value < 0.05).

#### 2.3.2 Bayesian *t*-test

The BMap was computed by performing a voxel-wise Bayesian one-sample *t*-test. Bayesian hypothesis testing does not provide a p-value, since it relies on assumptions different from those of frequentist statistics (Gelman and Shalizi, 2013, Kruschke and Liddell, 2018). In particular, Bayesian statistics assume parameters to be randomly distributed, while frequentists consider them to be fixed (Woolrich 2012). For this reason, the problem of type I error inflation in multiple comparisons is mitigated in a Bayesian framework, alleviating the strong need to perform correction for multiple comparisons (Han and Park, 2018, Gelman et al., 2012). The Bayesian method starts by establishing a prior probability distribution P(H) for the parameter of interest, which is hypothetical or based on some *a priori* knowledge about the parameter. Then the distribution is updated with the observed data, yielding the posterior probability distribution P(H|data). The update is based on the Bayes Theorem and given by **Equation** (1):

(1) $$P\left(H|data\right)=\frac{P\left(data|H\right)}{P\left(data\right)}P\left(H\right)$$

where P(data|H) is the likelihood of observing the data if the hypothesis H is true. To perform hypothesis testing, after stating the null (H_0_) and alternative (H_1_) hypotheses, their posterior probability distributions P(H_0_|data) and P(H_1_|data) can be calculated. Those allow for the marginal likelihoods P(data|H_0_) and P(data|H_1_) to be obtained. The ratio of the marginal likelihoods is called Bayes Factor (BF) (**Equation** (2)):

(2) $${\mathrm{BF}}_{10}=\frac{P(data|H_{1})}{P(data|H_{0})}$$

and it expresses how strongly the observed data supports the alternative hypothesis. BF_10_ ≥3 indicates moderate positive evidence in favor of the alternative hypothesis (Rouder et al., 2009, Wei et al., 2022) and BF_10_ ≥10 is usually chosen as a threshold indicating strong positive evidence supporting the alternative hypothesis (Kruschke and Liddell 2018).

In this study, the chosen null and alternative hypotheses were the same as used in the *t*-test and Wilcoxon test i.e. improvement <75 % and ≥75 % respectively. The prior probability distribution was updated with the data employing a Markov chain Monte Carlo method. Finally, each voxel was assigned a BF_10_ indicating how strongly the improvement data associated with that voxel would support the alternative hypothesis. Voxels with BF_10_ ≥10 were classified as belonging to the PSS.

The prior distribution was modelled as a normal distribution. This choice was made after conducting a prior sensitivity analysis on a sample of voxels, to estimate the influence of different prior distributions (normal, Cauchy and Student T) on the posterior distribution. The normal distribution is symmetric with defined mean and variance, the Cauchy distribution is heavy-tailed with undefined mean and variance, and the Student T distribution, with heavier tails than the normal, is particularly suited for small sample hypothesis testing. The three distributions were evaluated with metrics proposed in (Depaoli, Winter, and Visser 2020): model convergence assessment, posterior distribution visual inspection, comparison of posterior estimates and comparison of BF values. A more detailed explanation of these metrics is reported in Supplementary material. Examples of sensitivity analysis results are reported in Table S1 and Fig. S1. The sensitivity analysis showed that the posterior estimate was robust to the choice of normal distribution as the prior.

#### 2.3.3 Linear Mixed Model

The LMMMap was obtained by computing a Linear Mixed Model for each voxel as in (Vogel et al. 2024). The model identifies the voxels in which an increase in EF causes an increase in symptom improvement while considering the data variability due to the patient factor. Symptom improvement is, thus, the dependent variable, the EF norm is a fixed effect, and the patient is a random effect with variable slopes and intercepts across patients. P-values and coefficients of the model slopes were computed for each voxel. The PSS was determined by selecting only the voxels showing a significant positive relationship (positive slope) between EF norm and symptom improvement (0 ≤ p < 0.05).

#### 2.3.4 Type I error correction

Two correction methods were applied to the TMap and the WMap: a False Discovery Rate (FDR) approach (Benjamini and Hochberg 1995) and nonparametric permutations (Eisenstein et al. 2014). Contrary to the FDR approach, nonparametric permutations generate a summary statistic for the whole image, indicating whether to accept or reject the computed significant cluster. Therefore, a scaling of the permutation analysis from the image to voxel level was implemented. This voxel-wise permutations-based correction was inspired by (Nichols and Holmes 2001). Improvement values were permuted 200 times between the DBS leads and within the leads, resulting in 200 permuted datasets. Each permuted dataset was used to generate a probabilistic map. The p-values assigned to the same voxel across the permuted maps were compared to the p-value taken by the voxel in the true map. The corrected p-value for the voxel was computed as the percentage of permuted p-values lower or equal than the true p-value. Voxels with a corrected p-value ≥0.05 were discarded from the map. A graphical representation of the method is reported in Fig. 2**B**. With these two correction approaches TFDRMap, WFDRMap, TPermMap and WPermMap were obtained. The impact of the chosen number of permutations on the corrected cluster was also investigated. To do so, the voxel-wise permutation correction process described above was repeated with a number of permutations varying between 100 and 1000 in steps of 100. The obtained volumes were compared in terms of the number of voxels discarded by the correction and pairwise centroid distances. The results, reported in Fig. S2**,** showed that the number of permutations did not impact the extracted PSS In consequence for this work, it was chosen to use 200 permutations given the lower computational time.

#### 2.3.5 Masking and clustering

Three additional maps were created for masking the computed PSMs: a patient frequency map (nPatMap) reporting the number of patients in which a voxel was activated and a stimulation frequency map (nMap) indicating the number of stimulations activating each voxel. In the latter, the voxel average improvement score was weighted by the ratio between EF norm and current amplitude (Nordin et al. 2022), to penalize stimulations with higher amplitude. Voxels activated in less than 25 % of the patients or encompassed by a number of VTAs lower than 10 % of the maximum nMap value were discarded from the PSMs. Moreover, significant voxel clusters including less than 8 voxels (1 mm^3^) were discarded.

### 2.4 Probabilistic Sweet Spots comparison

A method for PSM generation was deemed reliable if it extracted PSS representative of clinical improvement and demonstrated robustness to outliers. To achieve this, the PSS were first compared in terms of size and anatomical location (topography). Secondly, the correlation between PSS stimulation and symptom improvement was calculated in a leave-one-out cross-validation fashion. Finally, the volumetric and positional consistency of PSS computed from the leave-one-out datasets were assessed. As each dataset consists of data from n − 1 patients (where n represents the total number of patients), the PSS calculated from these datasets serves as a valid indicator of the method's robustness to potential outliers.

#### 2.4.1 Volumetric and topographical analysis

For the topographical analysis the PSS, computed in the cohort-specific template space, were registered to the DB-MA (Deep Brain – MRI Architecture) atlas (Lemaire et al. 2024). The DB-MA atlas, among other datasets, comprises a T1 MR anatomical brain template and 118 labelled deep brain structures, which were used for the PSS visualization and anatomical localization. A correspondence between the anatomical nomenclature relevant for this study used in the DBMA and that proposed by Hassler (Hassler, Mundinger, and Riechert 2013) is provided in Table S2. The anatomical positions of the PSS were estimated by calculating the relative distance between their centroids and the ones of Vim and STN (Subthalamic Nucleus). Vim was selected because it is the standard target of DBS for tremor, and STN was chosen as an emblematic landmark within the subthalamus. The PSS volumes were calculated, together with their pairwise intersection, computed with the Dice coefficient.

#### 2.4.2 Correlation with improvement

The PSS is the brain area leading to the highest symptom improvement when stimulated. To verify the association of the extracted clusters with symptom improvement, the overlap between the PSS and each VTA (binarized by thresholding the electric field at 0.2 V mm^−1^) was computed. The overlap was calculated with the Dice coefficient. Each overlap value was coupled with the symptom improvement value associated with the VTA. The Spearman’s correlation coefficient between overlap and improvement was then calculated. To avoid biased correlation coefficients due to the usage of the same VTAs during PSS computation and verification, a leave-one-out cross-validation approach was applied. At each iteration VTAs from n-1 patients (where n is the total number of patients, here 23) were used to generate the PSS. The VTAs of the left-out patient were used to validate the obtained PSS. To minimize the influence of a limited number of points with high overlap on the correlation analysis, only those with overlap values below the 95th percentile of the distribution were included in the calculation.

#### 2.4.3 Consistency evaluation

The PSS generated during the leave-one-out cross-validation were also used to evaluate the method’s robustness to changes in the patient composition of the dataset. Firstly, geometric variability measures were computed within the methods. For each method, the PSS yielded by the iterations of the leave-one-out cross-validation scheme were used to calculate the following metrics:

- Volume coefficient of variation (**Equation** (3)):

(3) $$\mathrm{CV}=\frac{\mathrm{std}(volumes)}{\mathrm{mean}(volumes)}\times 100$$

- Pairwise Dice coefficient.
- Pairwise distance between centroids. The distance was normalized by the largest PSS diameter in the pair to avoid penalizing bigger PSS with respect to smaller ones.

Secondly, the distributions of variability scores were statistically compared between methods. This was possible only for the pairwise Dice coefficients and pairwise normalized centroids distances, since the volume coefficient of variation provided only one value per method. The values obtained were compared with one-way ANOVA and Tukey post-hoc testing, if some significant difference arose in the ANOVA. If the ANOVA assumptions were not met a Kruskal-Wallis test was applied.

## 3 Results

Probabilistic sweet spots were computed by applying four different statistical tests and two type I error corrections. This resulted in six distinct PSS, referred to as TFDR PSS, WFDR PSS, TPerm PSS, WPerm PSS, B PSS and LMM SS. This section reports the outcomes of the comparison between those six PSS with the methods described in section 2.4.

### 3.1 Volumetric and topographical analysis

**Fig. 3:**
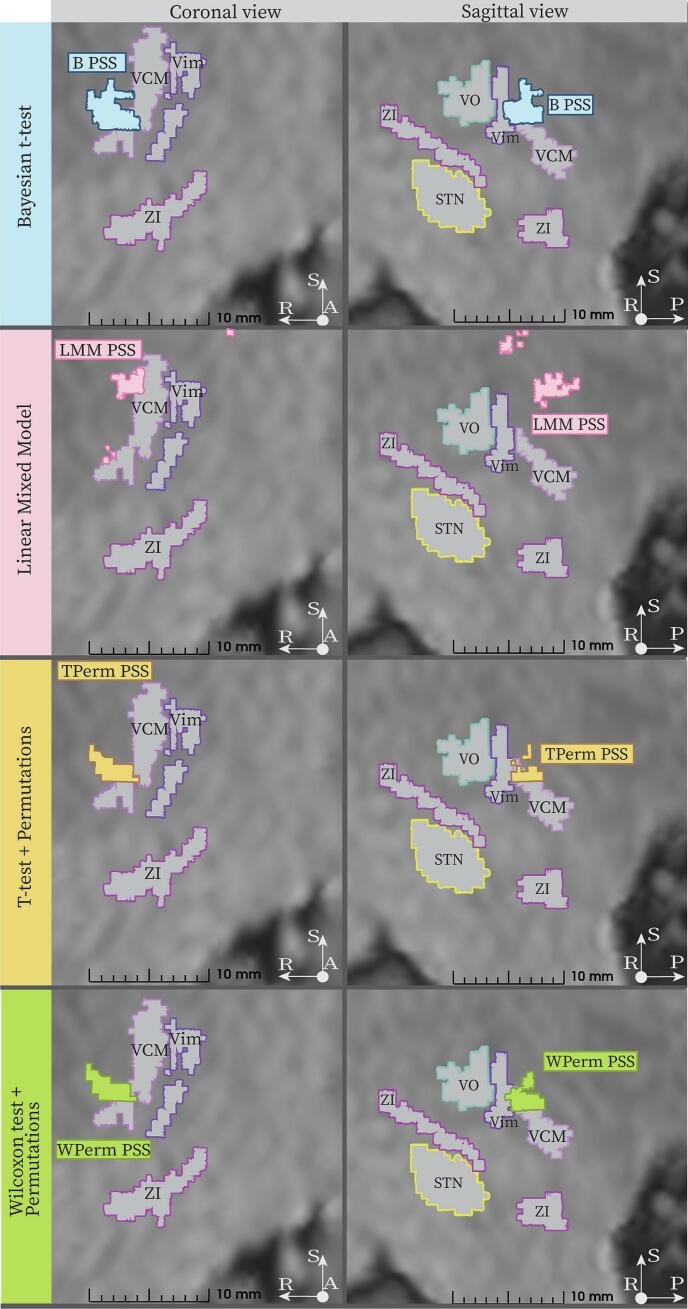
Probabilistic Sweet Spots and deep brain anatomical structures. Left hemisphere PSS are visualized in the DB-MA atlas (Lemaire et al. 2024). Going from top to bottom the PSS obtained with the Bayesian *t*-test (light blue), Linear Mixed Effect Model (pink), *t*-test (corrected with voxel-wise permutations, yellow) and Wilcoxon test (corrected with voxel-wise permutations, light green) are visualized in coronal and sagittal view. It was not possible to obtain Probabilistic Sweet Spots with the *t*-test and the Wilcoxon test corrected with FDR since no voxel survived the correction. Relevant deep brain structures are shown with grey filling and colored outline while the PSS are filled with the colors mentioned above. Abbreviations: VO: Ventrooral nucleus of Thalamus, VCM: Ventrocaudal medial nucleus of Thalamus, STN: Subthalamic Nucleus, ZI: Zona Incerta, Vim: Ventrointermediate nucleus of Thalamus.

Fig. 3 shows the PSS obtained from the entire dataset for the left hemisphere. The PSS are presented in the DB-MA atlas (Lemaire et al. 2024) with some relevant labelled deep brain structures.

The B PSS, TPerm PSS and WPerm PSS were located postero-medially to Vim and superiorly to STN and ZI (Zona Incerta), partially encompassing VCM-nuT (Ventrocaudal medial nucleus of thalamus). The LMM PSS was composed of two clusters located posteriorly and superiorly to Vim. The relative centroid distances between PSS and Vim and STN are reported in Table 2.

**Table 2:** Relative distances in mm of PSS centroids from centroids of average Vim and STN of the DB-MA atlas. Abbreviations: med. = medial, lat. = lateral, sup. = superior, inf. = inferior, post. = posterior, ant. = anterior.

	Distance to Vim [mm]			Distance to STN [mm]		
B PSS	3.88 med.	1.33 post.	1.45 inf.	0.81 med.	6.97 post.	8.22 sup.
LMM PSS	1.22 med.	1.38 post.	4.77 sup.	1.85 lat.	6.72 post.	14.44 sup.
TPerm PSS	4.23 med.	1.15 post.	1.75 inf.	1.17 med.	6.79 post.	7.92 sup.
WPerm PSS	4.18 med.	1.08 post.	1.68 inf.	1.12 med.	7.02 post.	7.99 sup.

**Fig. 4:**
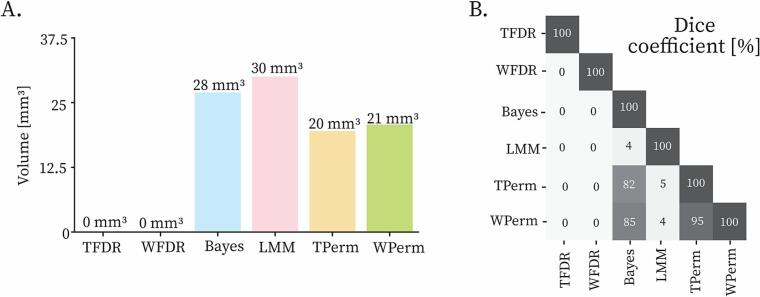
Bar plots with PSS volumes (A) and pairwise intersections (B). The intersections between PSS are calculated with Dice coefficients.

No voxel passed the FDR correction; therefore, it was not possible to obtain a TFDR PSS and a WFDR PSS. The surviving PSS volumes were 28 mm^3^ (B SS), 30 mm^3^ (LMM SS), 20 mm^3^ (TPerm SS) and 21 mm^3^ (WPerm SS) (Fig. 4**A**). The B PSS, TPerm PSS and WPerm PSS had many voxels in common with pairwise Dice coefficients of 95 % (between TPerm PSS and WPerm PSS), 82 % (between B PSS and TPerm PSS) and 85 % (between WPerm PSS and B PSS). On the contrary, for the LMM PSS, the intersection with the other PSS had a Dice coefficient only of 4 or 5 % (Fig. 4**B**).

### 3.2 Correlation with improvement

**Fig. 5:**
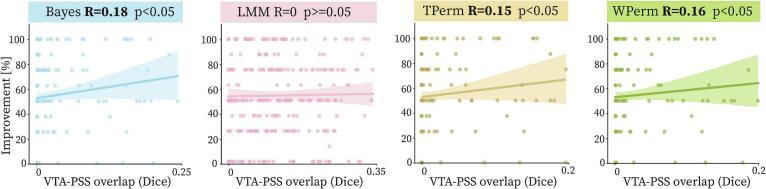
Linear correlation plots between VTA-PSS overlap and symptom improvement. Spearman’s correlation coefficients and statistical significance of the coefficient resulting from the leave-one-out cross-validation are reported for each method. Significant coefficients are shown in bold. The VTA-PSS overlap was calculated as Dice coefficient. The faded area around the regression line fit to the data shows the 95% confidence interval. Points with VTA-PSS overlap higher than the 95th percentile of the distribution were excluded from the calculation. TFDR and WFDR provided a PSS in only few iterations of the leave-one-out and were therefore excluded from the analysis.

The PSS were validated by calculating their correlation with symptom improvement in a leave-one-out cross-validation scheme. The TFDR and WFDR methods yielded PSS in only 5 and 4 of the leave-one-out iterations, respectively. This limited representation would have resulted in fewer data points and greater variability in the outcomes. Therefore, to ensure a fair comparison, TFDR and WFDR were excluded from subsequent analyses. Only points with overlap values below the 95th percentile of the distribution were included in the calculation. Considering this correction, the TPerm, WPerm and B generated PSS significantly (p < 0.05) correlated with improvement, with a Spearman’s correlation coefficient of 0.15, 0.16 and 0.18 respectively. LMM PSS did not have a significant correlation with improvement. The average results from the leave-one-out cross-validation are shown in Fig. 5. Fig. S3 shows the correlation results obtained by considering all the data points (e.g. also the ones with higher Dice coefficients).

### 3.3 Consistency

The PSS computed in the iterations of the leave-one-out cross-validation were geometrically compared in order to evaluate the consistency of each method when used on datasets composed of partially different patient groups. Also in this case, for the reasons mentioned above, the analysis was limited to B, LMM, WPerm and TPerm.

**Fig. 6:**
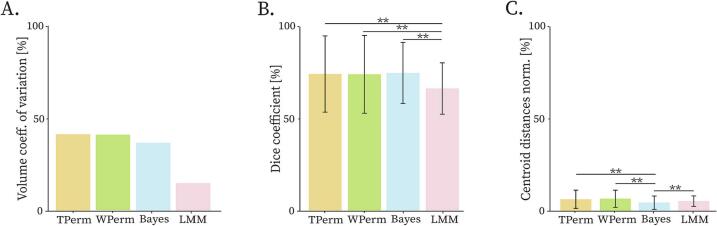
PSS consistency evaluation for all the methods. Going from left to right the bar plots show the volume coefficient of variation (in percentage) (A), the Dice coefficient (in percentage) (B) and the centroid distances normalized by the largest diameter (in percentage) (C). A consistent PSS (method robust to outliers) would be characterized by low volume coefficient of variation, high Dice coefficients and low centroid distances. Since the Dice coefficients and centroid distances were calculated in a pairwise fashion, the bars indicate the average values and standard deviations. TFDR and WFDR provided a PSS in only few iterations of the leave-one-out and were therefore excluded from the analysis. Statistically significant differences are indicated by the asterisks (* if p < 0.05, ** if p < 0.01 and *** if p < 0.001).

The volume coefficients of variation (Fig. 6**A**) were 34.62 % (B PSS), 38.97 % (TPerm PSS), 30.14 % (WPerm PSS), 14.17 % (LMM PSS). Pairwise Dice coefficients (mean ± std) (Fig. 6**B**) were 75.66 ± 16.76 % (B PSS), 75.1 ± 20.91 % (TPerm PSS), 74.90 ± 21.34 % (WPerm PSS), 67.01 ± 14.1 % (LMM PSS). B PSS, TPerm PSS and WPerm PSS had significantly higher pairwise Dice coefficients than LMM PSS (p < 0.01). The pairwise normalized centroid distances (mean ± std) (Fig. 6**C**) were 4.70 ± 3.50 % (B PSS), 6.47 ± 4.77 % (TPerm PSS), 6.73 ± 4.52 % (WPerm PSS), 5.48 ± 2.73 % (LMM PSS). B PSS had the lowest pairwise normalized centroid distances (p < 0.01).

## 4 Discussion

In this work, the impact of six statistical tests and correction combinations on the extraction of tremor PSS in DBS was assessed. The Bayesian *t*-test, along with voxel-wise permutation corrections applied to both the *t*-test and Wilcoxon test, showed the most promising results in pinpointing brain regions most associated with symptom improvement. In the last decade, several studies have tackled the challenge of probabilistic mapping of DBS therapeutic and adverse effects (Elias et al., 2021, Reich et al., 2019, Eisenstein et al., 2014, Butson et al., 2010, Akram et al., 2017, Vogel et al., 2024). Various workflows have been implemented and applied to different datasets and patient cohorts. However, the diversity of underlying data hinders a systematic methods comparison to assess their reliability and validity. An additional difficulty is posed by the absence of an established ground truth for DBS PSS. Because of these factors, the impact of methodological choices on the studies’ results is often overlooked. Few recent studies (Dembek et al., 2022, Nordin et al., 2022) have addressed this topic. (Nordin et al. 2022) investigated the influence of the data type (clinical vs screening data), clustering methods, sampling resolution of the electric field and weighting functions on the obtained PSMs. (Dembek et al. 2022) compared PSS generated with five voxel-wise methods using *in silico* data. Three of those methods were based on statistical testing and used either *t*-tests or Wilcoxon tests. The concordance of the computed sweet spots with the sensorimotor STN, chosen as ground truth, was calculated with the Dice coefficient. Two statistics-based methods showed higher Dice coefficients and higher explained variance when predicting improvement. The study, however, also highlighted variability in the obtained results. Going in the same direction, this work focused on voxel-wise statistical testing approaches, given their broad usage in the latest years (Reich et al., 2019, Nguyen et al., 2019, Neudorfer et al., 2021, Nowacki et al., 2022, Nowacki et al., 2020, Dembek et al., 2019). The aim was to assess the impact of the chosen statistical test and, if needed, type I error correction on the extracted Probabilistic Sweet Spots in DBS for ET patients. A secondary objective was to determine whether one of the approaches would be more reliable than the others in terms of correlation between the obtained cluster and symptom improvement, and results robustness to differences in patient datasets. The investigated statistical tests were the *t*-test, Wilcoxon test, Linear Mixed Model and Bayesian *t*-test. The first two were corrected with an FDR and a permutation-based approach, resulting in 6 different PSS. This study also introduced some novelties in the probabilistic stimulation mapping scenario. To our knowledge, our consortium is the only one working with intra-operative stimulation data. Furthermore, this work is the first to introduce Bayesian statistical testing and voxel-wise nonparametric permutation corrections to map DBS PSS.

### 4.1 Topography and volumetric differences

Different methods led to the extraction of PSS with variable sizes, as also observed in (Dembek et al. 2022). TFDR and WFDR did not provide a PSS. The highest similarity was shown by the B PSS, the TPerm PSS and the WPerm PSS. All three PSS centroids were located posteriorly, medially and inferiorly to Vim, in an area commonly known as Posterior Subthalamic Area (PSA). This result is in agreement with most of the tremor suppression hotspots already presented in literature. (Al-Fatly et al., 2019, Vogel et al., 2024, Nowacki et al., 2022, Kübler et al., 2021) pinpointed, in fact, a tremor sweet spot in the PSA. On the other hand (Dembek et al. 2017) and (Elias et al. 2021) identified high improvement areas in the ZI and in the anterior border of Vim, respectively. It should also be noted that variations in sweet spot locations between patient cohorts may also partly reflect differences in surgical targeting strategies and electrode placement techniques.

Despite the anatomical proximity, the three PSS showed a maximum volumetric difference of 10  mm^3^, which can be relevant if compared to the sizes of deep brain structures (e.g. Vim volume in DB-MA atlas ∼ 163 mm^3^).

The biggest difference was observed between the LMM PSS and all the other PSS, particularly in terms of anatomical location (LMM PSS superior to Vim). This could be explained by considering the different hypotheses underlying the methods. TPerm, WPerm and B PSS include voxels with an average improvement significantly higher than a chosen threshold (75 % in this work). On the other hand, the LMM identifies voxels where a significant positive relationship between the EF norm and improvement is observed, without posing constraints on the average improvement score of the voxel. This can result in the selection of different voxels. Such results seem to contrast with our previous ones (Vogel et al. 2024), where a Linear Mixed Model was also implemented. In that study, however, a more comprehensive dataset was used, featuring several stimulations per patient (>200 data points per patient vs. ∼ 30 data points per patient in this study). Additionally, improvement scores were derived from accelerometer measurements, providing both negative values and continuous scale. This suggests that the LMM may be less appropriate for datasets with limited data points per patient, few improvement categories or only optimal stimulation settings.

### 4.2 Correlation with improvement

We investigated whether the computed PSS were representative of clinical improvement by calculating Spearman’s correlation coefficients between the overlap of each VTA in the dataset and the PSS, and the improvement associated with the VTA. To avoid biases due to the usage of the same data to generate and then validate the PSS, a leave-one-out cross-validation scheme was followed. TFDR and WFDR provided PSS in only few iterations, so they were not considered in the comparison. The LMM PSS did not have a significant correlation with improvement. Contrarily, B PSS, TPerm PSS, and WPerm PSS had significant but low correlation coefficients, with values ranging between 0.15 and 0.18. Furthermore, with the exclusion of data points with overlap values above the 95th percentile, the Dice coefficient between the VTAs and the PSS ranged from 0 to 0.3, reflecting limited spatial overlap between the volumes. This may be attributed to the relatively small extent of the identified PSS compared to the VTAs. Additionally, the limited sample size likely amplified the influence of outliers — such as patients exhibiting high improvement in regions distant from the majority—on the correlation analysis.

Moderate R-values were reported by (Neudorfer et al. 2023) who also calculated overlap-improvement Spearman’s correlation coefficients. Higher coefficients (0.65) were instead obtained by (Elias et al. 2021). (Dembek et al. 2019), on the other hand, calculated a linear regression model between overlap and improvement. In these studies the overlap was calculated with different strategies: (Dembek et al. 2019) computed it as the percentage of PSS volume, (Elias et al. 2021) as a clinically weighted number of voxels and (Neudorfer et al. 2023) introduced a “sweet spot score” based on the average improvement value in the overlap area. In this work, the first and last calculation methods were applied, yielding comparable correlation coefficients (Fig. S4, S5). The second method was not applicable since the output of the workflow in this study was a binary PSS. The final results are presented as Dice coefficient values. The significant but low correlation coefficients denote that the overlap between VTA and the sweet spot is probably not the only biomarker of symptom improvement in DBS, requiring further investigation. In fact, it has been shown that improvement prediction models can benefit from the inclusion of clinical features (Shamir et al. 2015), side effects-based features (Boutet et al. 2021) and electrophysiology measurements (Shah et al. 2023).

### 4.3 Consistency

The methods were subsequently evaluated for consistency when applied to datasets with varying compositions. A reliable method should produce results that accurately reflect the input data while remaining robust to outliers. Therefore, adding or removing a single patient from the dataset is expected to result in minor variations in the average PSS, but these changes should not be substantial. To assess each method's sensitivity to dataset variations of one patient at a time the leave-one-out datasets were used. For each method, the geometric variability of the PSS calculated in each iteration of the leave-one-out cross-validation was analysed. B PSS, TPerm PSS and WPerm PSS had significantly higher pairwise Dice coefficients than the PSS obtained with LMM. B PSS had the significantly lowest pairwise normalized centroids distances, establishing it as the most robust method.

### 4.4 Discrete vs. Continuous data

In this study, the tremor improvement was assessed by the neurologist using a discrete rating scale, similar to what reported in (Dembek et al. 2017). Given the nature of the data, the most appropriate statistical test would have been the Wilcoxon test. Nevertheless, we also opted to include the *t*-test due to its widespread use in probabilistic mapping scenarios, the Bayesian *t*-test given its promising performance in fMRI studies, and the Linear Mixed Model based on its possibility to model patient dependency (Vogel et al. 2024). This decision was taken despite the fact that these methods are primarily designed for continuous data. The observed similarity in results between the Wilcoxon test, *t*-test, and Bayesian *t*-test suggests that both these last two can be effectively applied to discrete data in this context. In contrast, the Linear Mixed Model did not perform well when presented with discrete data. Importantly, our analysis indicates that the choice of correction method (False Discovery Rate vs. permutation-based corrections) has a more significant impact on the results than the choice between the Wilcoxon test or *t*-test.

### 4.5 Intra-operative vs post-operative stimulation data

Probabilistic mapping studies so far have exploited stimulation data collected post-operatively (e.g. clinical stimulation settings (Eisenstein et al., 2014, Nowacki et al., 2022), or monopolar review data (Dembek et al. 2017)). On the contrary, this study utilized data from intra-operative stimulation tests. Such tests offer the notable advantage of closer spacing of measurement positions, resulting in higher data density compared to stimulation tests from monopolar reviews. The findings demonstrate that intra-operative data are both suitable and informative for probabilistic mapping. Additionally, comparing the optimal stimulation areas derived from intra-operative and post-operative data within the same patient cohort could yield valuable insights.

## 5 Limitations and future steps

A primary limitation of this study is the sample size and the limited granularity of the improvement scores. While it provides a solid foundation and is representative for post-operative stimulation datasets obtained during monopolar reviews, more precise evaluations could be achieved with a larger dataset that includes additional stimulation tests per position and finer-grained scoring. Although the ratings were performed by the same experienced neurosurgeon (JJL), they inherently rely on subjective assessment, introducing some degree of rater-dependent variability. The use of validated rating protocols such as the one used in (Sandström et al., 2019, Hägglund et al., 2016) or objective measures such as accelerometer-based assessments (Shah et al. 2017) would be particularly beneficial in this context. Nonetheless, (Hariz et al. 2021) showed that even inter-rater variability is low for well-trained evaluators with long experience. Furthermore, increasing the number of patients would enhance the generalizability of the results and help determine whether certain methods are better suited for smaller or larger datasets. Additionally, the study did not incorporate data on the occurrence of side effects, which could offer valuable insights into the clinical relevance of the identified PSS. Finally, the correlation between the stimulated portion of the PSS and improvement was chosen as metric to assess the clinical effectiveness of the PSS. However, the low correlation coefficients suggest that more factors may come into play in determining outcomes of DBS. Future research should, thus, be oriented to the investigation of improvement biomarkers.

## 6 Conclusions

In this study we investigated the impact of six statistical tests and corrections combinations on the extraction of tremor PSS in DBS. Researchers working with probabilistic mapping should be aware that the chosen method strongly influences the extracted volume. The Bayesian *t*-test, newly introduced in the context of DBS, along with voxel-wise permutation corrections applied to both the *t*-test and Wilcoxon test, yielded the most promising results in pinpointing brain regions most associated with symptom improvement. These agree with findings from previous studies based on monopolar review stimulation data, supporting the relevance of PSMs computed from intra-operative stimulation data. The Bayesian *t*-test also demonstrated greater robustness to minor dataset variations, indicating its potential for future use in DBS research after further validation on more extensive datasets. We therefore recommend using either a Bayesian *t*-test, or a permutation-corrected analytical test (*t*-test or Wilcoxon test) for calculating PSMs based on post-operative or intra-operative optimal stimulation settings (∼10–20 stimulations per patient in each hemisphere). In contrast, Linear Mixed Models appear more suitable for datasets with a higher number of stimulations per patient (at least 100 data points per patient).

## CRediT authorship contribution statement

**Vittoria Bucciarelli:** Writing – original draft, Software, Methodology, Conceptualization. **Dorian Vogel:** Writing – review & editing, Methodology, Conceptualization. **Teresa Nordin:** Writing – review & editing, Methodology, Conceptualization. **Marc Stawiski:** Writing – review & editing, Data curation. **Jérôme Coste:** Writing – review & editing, Validation. **Jean-Jacques Lemaire:** Writing – review & editing, Validation. **Raphael Guzman:** Writing – review & editing, Supervision. **Simone Hemm:** Writing – review & editing, Supervision, Methodology, Funding acquisition, Conceptualization.

## Data Availability

Data will be made available on request.

## Footnotes

1 https://liu.se/en/article/ne-downloads.

2 https://www.slicer.org/.
