# Supplementary material for: Influence of statistical approaches on Probabilistic Sweet Spots computation in Deep Brain Stimulation for severe Essential Tremor
Source: Neuroimage Clin. 2025 Jun 7;47:103820. doi: 10.1016/j.nicl.2025.103820 (PMC12192744; doi:10.1016/j.nicl.2025.103820)
Supplement: Supplementary Data 1 [file mmc1.docx]

Supplementary material

# Bayesian sensitivity analysis

In Bayesian inference, prior knowledge (expressed as a prior distribution) is combined with evidence from the data (likelihood) to form the posterior distribution. The Bayesian t-test can be applied to distributions with small sample sizes and does not require the assumption of normality unlike other statistical tests (e.g. frequentist t-test) (Gelman and Shalizi 2013; Kruschke and Liddell 2018). However, the outcome of the Bayesian t-test can be influenced by the characteristics of the chosen prior distribution. Therefore, when the prior distribution of the data is not known *a priori*, it is possible to evaluate the influence of the prior choice on the posterior probability distribution. This procedure is called sensitivity analysis and can be used to inform the choice of the prior. In this work, the sensitivity analysis was performed calculating the metrics reported in (Depaoli, Winter, and Visser 2020) on a sample of voxels to choose between a normal, T-student or Cauchy prior distribution. Such metrics are:

- Model convergence evaluation by considering the R-hat and effective sample size (ESS) values. In particular, R-hat<1.01 and ESS>1000 indicate model convergence and enough precision in the chain.
- Posterior distribution visual inspection: The posterior distribution is the probability distribution that represents updated beliefs about an unknown parameter after observing new data. If the posterior distributions are very similar it means that the posterior estimate is robust to different priors (and therefore the different priors would yield approximately the same results).
- Percentage deviation in the average posterior estimate computation: a low percentage deviation means high robustness to prior. A posterior estimate is a summary value (mean in this case) derived from the posterior distribution. The percentage deviation estimates how much the average posterior estimate differs from the true value.
- Bayes Factor comparison and consequent voxel classification.

**Table S1** and **Figure S1** report example results obtained on some of the voxels involved in the sensitivity analysis. The data showed robustness to the prior choice, so a normal prior was chosen for the sweet spot calculation.

Table S1. Sensitivity analysis numerical metrics calculated for four example voxels and three prior distributions.

| Voxel 1 | ESS | R-hat | % dev. | BF |
| --- | --- | --- | --- | --- |
| Normal | 1388 | 1.0 | -0.018 | 0.04 |
| Cauchy | 1631 | 1.0 | 0.122 | 0.032 |
| T-student | 1691 | 1.0 | 0.11 | 0.041 |
| Voxel 2 | **ESS** | **R-hat** | **% dev.** | **BF** |
| Normal | 1860 | 1.0 | 0.08 | 0.0081 |
| Cauchy | 1846 | 1.0 | 0.25 | 0.007 |
| T-student | 1769 | 1.0 | 0.19 | 0.0083 |
| Voxel 3 | **ESS** | **R-hat** | **% dev.** | **BF** |
| Normal | 1607 | 1.0 | -0.026 | 0.56 |
| Cauchy | 1953 | 1.0 | 0.15 | 0.61 |
| T-student | 1593 | 1.0 | 0.18 | 0.59 |
| Voxel 4 | **ESS** | **R-hat** | **% dev.** | **BF** |
| Normal | 1503 | 1.0 | -0.36 | 4500 |
| Cauchy | 1836 | 1.0 | -0.52 | 3999 |
| T-student | 1824 | 1.0 | -0.31 | 799 |


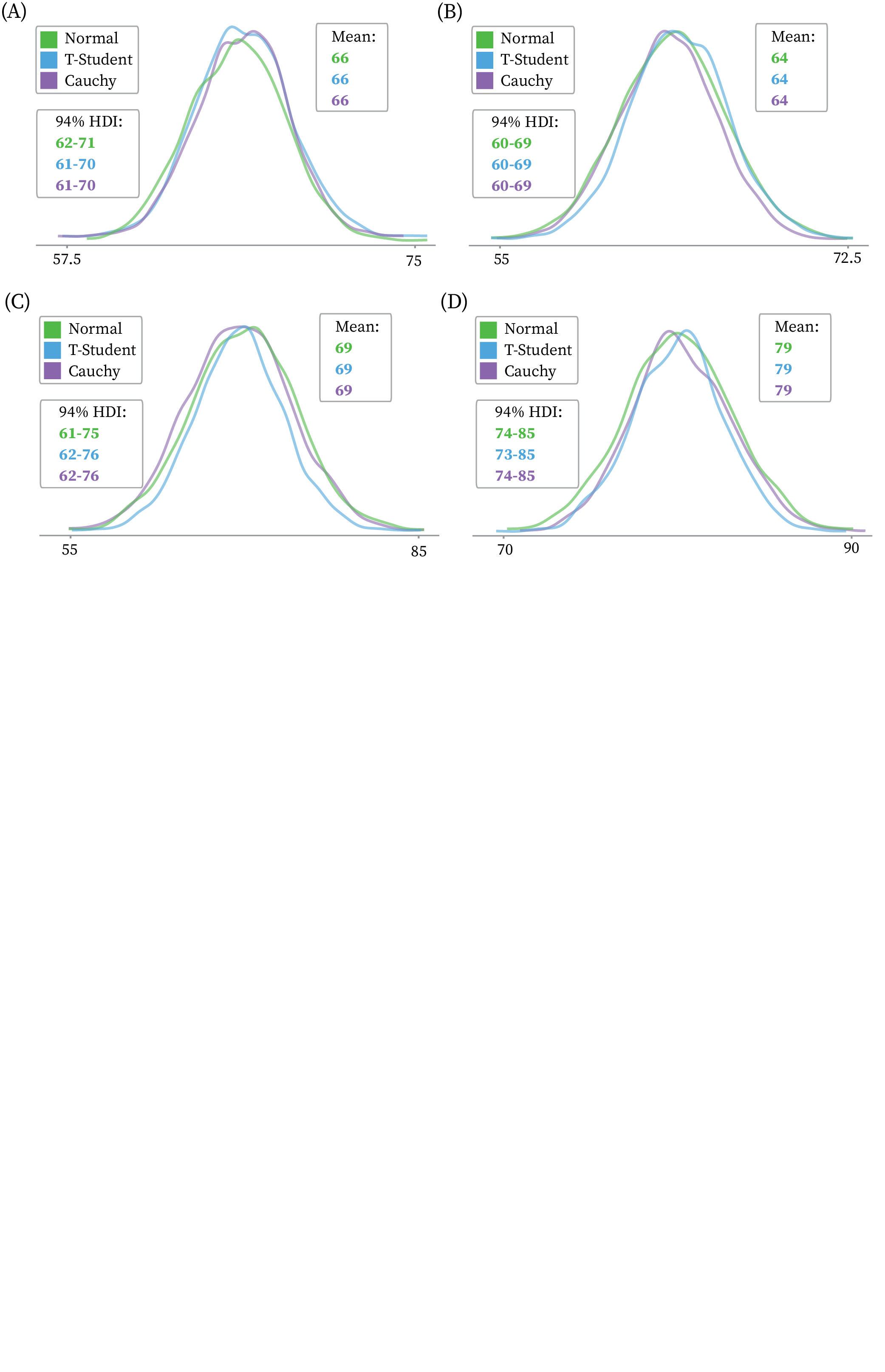


Figure S1. Posterior distributions plots for the normal, T-student and Cauchy prior for example voxel 1 (A), voxel 2 (B), voxel 3 (C) and voxel 4 (D). The mean values and 94% highest posterior density intervals (HDI) values are reported in the boxes.

# Voxel-wise nonparametric permutation approach: choice of permutations number

To investigate the impact of the chosen number of permutations on the corrected cluster the voxel-wise permutation correction process (applied to the t-test) was repeated with a number of permutations varying between 100 and 1000 in steps of 100. The obtained volumes were compared in terms of number of voxels discarded by the correction and pairwise centroid distances. Results are shown in **Figure S2**.


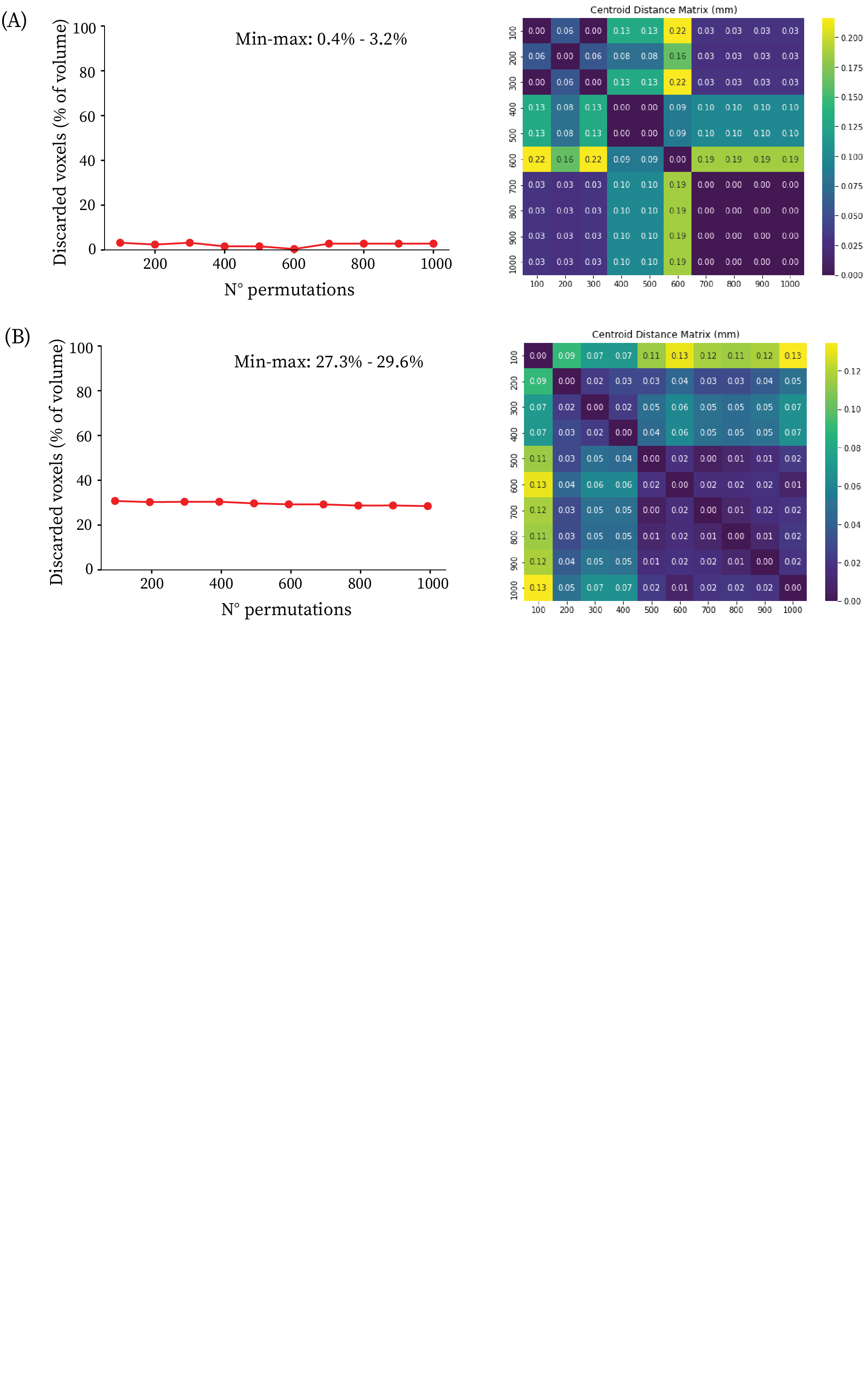


Figure S2. Percentage of discarded volume for each permutation number (100 to 1000 with steps of 100) (on the left) and pairwise centroid distances for each number of permutations (on the right).

# Calculation of correlation with improvement for the full dataset

To minimize the influence of a limited number of points with high overlap on the correlation analysis, only those with overlap values below the 95th percentile of the distribution were included in the calculation presented in the manuscript. Here **Figure S3** shows the results obtained on the full dataset, without exclusion of high overlap points.


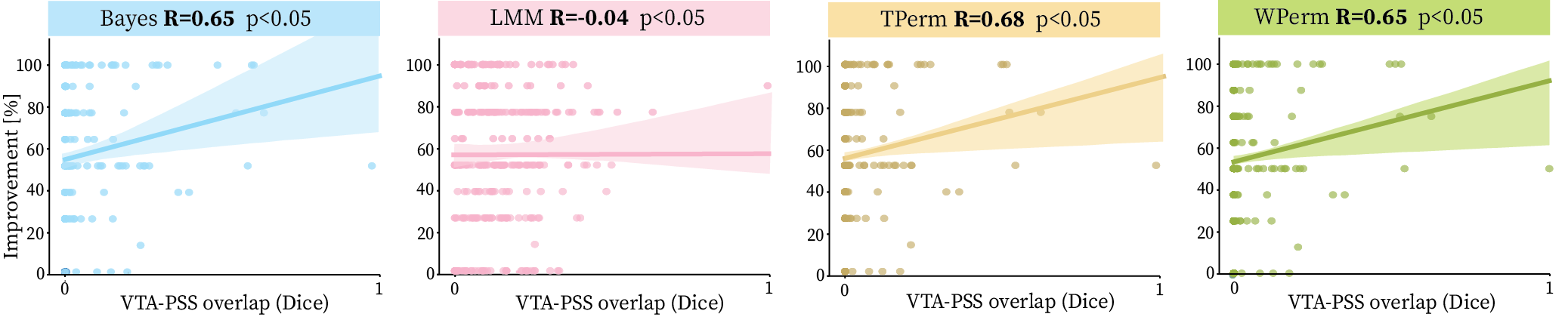


Figure S3. Spearman’s correlation coefficients and statistical significance of the coefficient resulting from the leave-one-out cross-validation are reported for each method. Significant coefficients are shown in bold. The VTA-PSS overlap was calculated as Dice coefficient. The faded area around the regression line fit to the data shows the 95% confidence interval. TFDR and WFDR provided a PSS in only few iterations of the leave-one-out and were therefore excluded from the analysis.

# Calculation of correlation with improvement with alternative overlap metrics

The Probabilistic Sweet Spots (PSS) association with clinical improvement was evaluated by calculating Spearman’s correlation coefficient between VTA-PSS overlap and improvement scores. In the manuscript, the VTA-PSS overlap was calculated as Dice coefficient. In addition, the overlap was also calculated as:

- Percentage of PSS volume as in (Dembek et al. 2019) with results reported in **Figure S4**
- “sweet spot score” as in (Neudorfer et al. 2023) with results reported in **Figure S5**


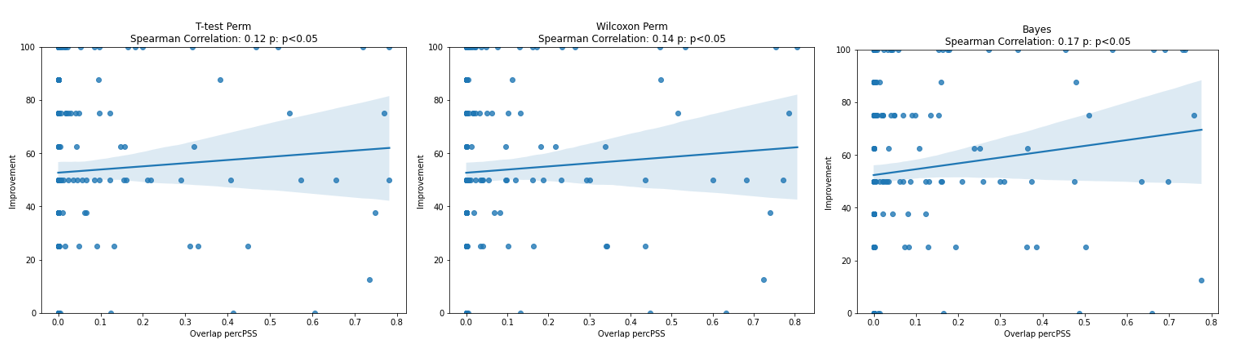


Figure S4. Linear correlation plots between VTA-PSS overlap and symptom improvement. The faded area around the regression line fit to the data shows the 95% confidence interval. Spearman’s correlation coefficients and p-values resulting from the leave-one-out cross-validation are reported for each method. The VTA-PSS overlap was calculated as percentage of sweet spot volume. Overlap values above the 95^th^ percentile were removed to avoid biasing of the correlation results.


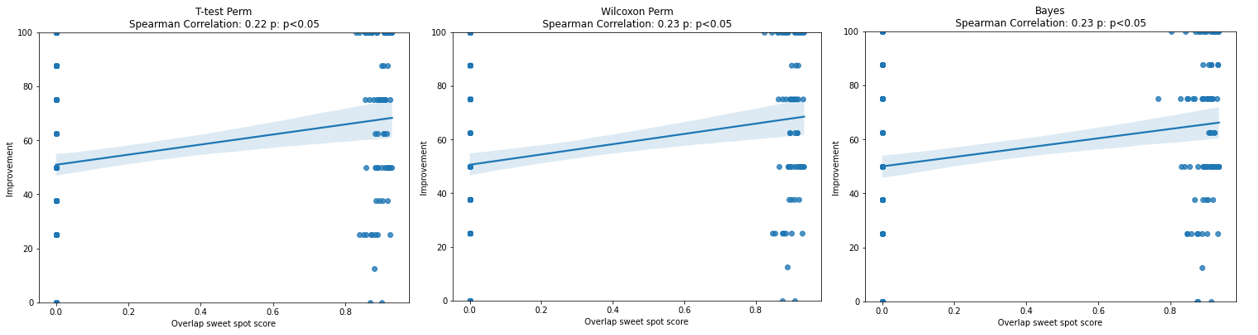


Figure S5. Linear correlation plots between VTA-PSS overlap and symptom improvement. The faded area around the regression line fit to the data shows the 95% confidence interval. Spearman’s correlation coefficients and p-values resulting from the leave-one-out cross-validation are reported for each method. The VTA-PSS overlap was calculated as sweet spot score (average improvement value in the voxels encompassed by both the VTA and the PSS). Overlap values above the 95^th^ percentile were removed to avoid biasing of the correlation results.

# Mapping of thalamic nomenclature from DBMA atlas to Hassler

Table S2. Correspondences of relevant deep brain structures nomenclatures between DBMA atlas (Lemaire et al. 2024) and Hassler nomenclature (Hassler, Mundinger, and Riechert 2013; Schaltenbrand 1977).

| **Full name** | **DBMA** | **Hassler** |
| --- | --- | --- |
| Ventrooral Anterior nucleus of Thalamus | VOA-nuT | V.o.a. |
| Ventrooral Posterior nucleus of Thalamus | VOP-nuT | V.o.p. |
| Ventrooral Mediodorsal nucleus of Thalamus | VOM-nuT | V.o.m. |
| Ventrointermediate nucleus of Thalamus | Vim | V.im. |
| Ventrocaudal medial nucleus of thalamus | VCM-nuT | V.c.i. |
| Zona Incerta | ZI | Z.i. |
| Subthalamic Nucleus | STN | S.th. |

# References

Dembek, T.A., Jan Roediger, Andreas Horn, Paul Reker, Carina Oehrn, Haidar S. Dafsari, Ningfei Li, et al. 2019. “Probabilistic Sweet Spots Predict Motor Outcome for Deep Brain Stimulation in Parkinson Disease.” *Annals of Neurology* 86 (4): 527–38. https://doi.org/10.1002/ana.25567.

Depaoli, Sarah, Sonja D. Winter, and Marieke Visser. 2020. “The Importance of Prior Sensitivity Analysis in Bayesian Statistics: Demonstrations Using an Interactive Shiny App.” *Frontiers in Psychology* 11 (November):608045. https://doi.org/10.3389/fpsyg.2020.608045.

Gelman, Andrew, and Cosma Rohilla Shalizi. 2013. “Philosophy and the Practice of Bayesian Statistics.” *British Journal of Mathematical and Statistical Psychology* 66 (1): 8–38. https://doi.org/10.1111/j.2044-8317.2011.02037.x.

Hassler, R., F. Mundinger, and T. Riechert. 2013. *Stereotaxis in Parkinson Syndrome: Clinical-Anatomical Contributions to Its Pathophysiology*. Springer Science & Business Media.

Kruschke, John K., and Torrin M. Liddell. 2018. “The Bayesian New Statistics: Hypothesis Testing, Estimation, Meta-Analysis, and Power Analysis from a Bayesian Perspective.” *Psychonomic Bulletin & Review* 25 (1): 178–206. https://doi.org/10.3758/s13423-016-1221-4.

Lemaire, Jean-Jacques, Rémi Chaix, Aigerim Dautkulova, Anna Sontheimer, Jérôme Coste, Ana-Raquel Marques, Adrien Wohrer, et al. 2024. “An MRI Deep Brain Adult Template With An Advanced Atlas-Based Tool For Diffusion Tensor Imaging Analysis.” *Scientific Data* 11 (1): 1189. https://doi.org/10.1038/s41597-024-04053-x.

Neudorfer, Clemens, Konstantin Butenko, Simon Oxenford, Nanditha Rajamani, Johannes Achtzehn, Lukas Goede, Barbara Hollunder, et al. 2023. “Lead-DBS v3.0: Mapping Deep Brain Stimulation Effects to Local Anatomy and Global Networks.” *NeuroImage* 268 (March):119862. https://doi.org/10.1016/j.neuroimage.2023.119862.

Schaltenbrand, Georg. 1977. *Atlas for Stereotaxy of the Human Brain, with an Accompanying Guide*. Second, Revised and Enlarged edition.. Stuttgart: G. Thieme.
